# Renal potassium handling in carriers of the Gly40Ser mutation of the glucagon receptor suggests a role for glucagon in potassium homeostasis

**DOI:** 10.14814/phy2.13661

**Published:** 2018-04-19

**Authors:** Lise Bankir, Antonio Barbato, Ornella Russo, Gilles Crambert, Roberto Iacone, Nadine Bouby, Ludovica Perna, Pasquale Strazzullo

**Affiliations:** ^1^ INSERM Unit 1138 Centre de Recherche des Cordeliers Paris France; ^2^ Université Pierre et Marie Curie Paris France; ^3^ Department of Clinical Medicine and Surgery Federico II University Medical School Naples Italy; ^4^ CNRS ERL8228 Metabolism and Renal Physiology Paris France

**Keywords:** Glucose, Insulin, Mendelian randomization, Sodium, TTKG, Urea

## Abstract

Plasma potassium concentration (P_K_) is tightly regulated. Insulin is known to favor potassium entry into cells. But how potassium leaves the cells later on is not often considered. Previous studies in rats showed that glucagon infusion increased urinary potassium excretion dose‐dependently and reversibly. This prompted us to investigate the possible influence of glucagon on potassium handling in humans. We took advantage of the Gly40Ser mutation of the glucagon receptor (GR) that results in a partial loss of function of the GR. In the Olivetti cohort (male workers), 25 subjects who carried this mutation were matched 1:4 to 100 noncarriers for age and weight. Estimated osmolarity of serum and 24‐h urine (S_osm_ and U_osm,_ respectively) was calculated from the concentrations of the main solutes: [(Na+K)*2 + urea (+glucose for serum)]. Transtubular potassium gradient (TTKG), reflecting the intensity of K secretion in the distal nephron, was calculated as [(urine K/serum K)(U_osm_/S_osm_)]. There was no significant difference in serum K, or 24‐h urine urea, Na and K excretion rates. But urine K concentration was significantly lower in carriers than in noncarriers. Means (interquartile range): 38 (34–43) versus 47 (43–51) mmol/L, *P* = 0.030. TTKG was also significantly lower in carriers: 4.2 (3.9–4.6) versus 5.0 (4.7–5.2), *P* = 0.015. This difference remained statistically significant after adjustments for serum insulin and 24‐h Na and urea excretions. These results in humans suggest that glucagon stimulates K secretion in the distal nephron. Thus, in conjunction with insulin, glucagon may also participate in K homeostasis by promoting renal K excretion.

## Introduction

The regulation of plasma potassium concentration (P_K_) is assumed to depend mainly on aldosterone. However, aldosterone has an opposite influence on the excretion rates of potassium and sodium and thus cannot alone regulate the plasma level of each of these two cations independently. Insulin may play an additional role. Insulin release is stimulated after a large oral intake or infusion of potassium, and this hormone allows potassium entry into cells (mostly hepatocytes and muscle cells), thus attenuating the rise in P_K_. But potassium cannot be stored in these cells permanently. To our knowledge, how potassium is subsequently released by these cells has never been addressed thus far. Nevertheless, since potassium excretion increases more rapidly after the ingestion or infusion of a potassium load than what would be expected from a steroid‐dependent mechanism, a few authors have postulated the existence of an aldosterone‐independent kaliuretic factor (Rabinowitz 1991; Gumz et al. 2015).

During renal clearance studies in rats, we observed that an i.v. infusion of glucagon induced a marked increase in urinary potassium excretion that was dose‐dependent and reversible (Ahloulay et al. 1995). This suggested that glucagon could facilitate the excretion of potassium by stimulating potassium secretion in the collecting duct, and thus could reduce P_K_ and allow the progressive release of potassium from the cells where it was temporarily stored under the influence of insulin. As glucagon and insulin play opposite roles in glucose homeostasis, it is conceivable that they may also play opposite roles in potassium homeostasis. Several arguments supporting this hypothesis have been described in a recent review (Bankir et al. 2016).

A polymorphic variant of the glucagon receptor gene has been described. It is the result of a missense mutation leading to the substitution of a serine for a glycine in the encoded protein (Gly40Ser) (Shiota et al. 2002; Barbato et al. 2003). This variant is present in 1–3% of Caucasian populations. The receptor protein expressing this variant is less sensitive to glucagon, as indicated by a lower production of cAMP by liver cell membranes in vitro (Hager et al. 1995; Hansen et al. 1996) and by a twofold lower rise in blood glucose in response to the systemic infusion of glucagon in individuals carrying the mutation (Tonolo et al. 1997).

Previous studies in the Olivetti cohort (including adult men workers of the Olivetti Company in southern Italy) have described that the Gly40Ser mutation of the glucagon receptor is associated with an increased risk of hypertension, an enhanced proximal tubular sodium reabsorption (Strazzullo et al. 2001) and with anthropometric indices of central adiposity (Siani et al. 2001).

In the present study, we analyzed data collected in the Olivetti participants to determine whether the carriers of the Gly40Ser mutation of the glucagon receptor exhibit an abnormal potassium handling. We hypothesized that a less sensitive glucagon receptor in the kidney might lead to a less efficient secretion of potassium in the collecting duct and thus, to a less efficient urinary excretion of potassium.

## Subjects and Methods

The Olivetti Heart Study is an epidemiological investigation of cardiovascular risk factors carried out in southern Italy. The study started in 1975 with periodical follow‐up for 30 years and involved the Olivetti factories male workforce (Olivetti Heart Study official website: http://www.olivettiheartstudy.org). Most of the data presented in the present article were collected during the 2002–2004 follow‐up visit during which a total of 940 men (age range 25–75 years) were examined. The local Ethics Committee approved the study protocol, and participants provided informed written consent.

### Procedures

A detailed description of the procedures has been reported in previous papers (Cappuccio et al. 1993; Strazzullo et al. 2001; Galletti et al. 2007). The participants underwent anthropometric measurements, fasting blood tests, a fixed‐sequence questionnaire including demographic information, past medical history, and a food frequency questionnaire. Body weight and height were measured and the body mass index (BMI) was calculated as weight (kg) divided by the square of the height (m). The waist circumference was measured at the umbilicus level. Systolic and diastolic (phase V) blood pressure (SBP and DBP, respectively), and heart rate (HR) were taken three times, 2 min apart, with a random zero sphygmomanometer (Gelman Hawksley Ltd, Sussex, UK) and the average of the last two readings was used for SBP, DBP and HR.

A fasting venous blood sample was taken and the blood specimens were immediately centrifuged and stored at −70°C until analyzed. A 24‐h urine collection was obtained from each participant, on the day preceding the visit, for the measurement of sodium, potassium, urea and creatinine excretion. This urine was delivered to the laboratory for immediate storage of aliquot samples at −70°C until measurement.

The assessment of the Gly40Ser polymorphism of the glucagon receptor had been carried out on 971 out of 1075 participants at follow‐up visit in 1994–95, as described previously (Hager et al. 1995). The Gly40Ser variant was present in 37 subjects (3.8%). There was no subject homozygote for the mutation. The allele distribution followed the Hardy–Weinberg equilibrium. Of the original 971 participants, 812 (84%) were again seen at 2002–04 follow‐up examination. For the purpose of this analysis, a complete database for the serum and urinary potassium parameters at the 2002–2004 follow‐up examination was available for 25 carriers of the G allele. We then selected in the same cohort the highest possible number of age‐ and weight‐matched CC controls for each of the 25 carriers of the G allele. This resulted in 4 CC noncarriers for each of the G allele carriers.

### Laboratory measurements and calculations

Serum glucose concentration and serum and urinary urea concentration were measured using an automated analyser (Cobas‐Mira; Roche, Milan, Italy). Serum and urinary creatinine concentration was assessed by the picric acid colorimetric method (Jaffe‘s) using a Cobas‐Mira analyzer. Serum and urinary sodium and potassium concentrations (S_Na_, U_Na_, S_K_, and U_K_, respectively) were measured by atomic absorption spectrophotometry. Serum insulin concentration was measured by radioimmunoassay (Insulina Lisophase, Technogenetics, Milan, Italy).

The volume of the previous 24‐h urine collection was measured. Urine osmolarity (U_osm_ in mosm/L) was estimated by the following formula: (U_Na_ + U_K_) × 2 + U_urea_, where U_Na_, U_K_ and U_urea_ are the urinary concentrations of sodium, potassium, and urea, respectively, in mmol/L (Youhanna et al. 2017). Serum osmolarity (S_osm_) was calculated in the same way, with the addition of serum glucose concentration. The sodium and potassium excretion rates were calculated by multiplying their respective urinary concentration by the 24‐h urine volume.

The transtubular potassium gradient (TTKG) is an index used to quantify the intensity of active potassium secretion in the cortical collecting duct (West et al. 1986a). It is calculated by the formula TTKG = (U_K_/S_K_)/(U_osm_/S_osm_). It reflects how much potassium is concentrated in the urine above its plasma value, in excess of all osmoles.

### Statistical analysis

Results are expressed as means and 95 % confidence intervals (CI) unless otherwise indicated. Two‐sided *P* < 0.05 was considered statistically significant. The distributions of serum glucose and insulin deviated significantly from normality. Analysis of variance (ANOVA) was used to assess differences between group means for all normally distributed variables. The Mann–Whitney *U* test was used to evaluate statistical differences between groups when the variables were not normally distributed.

Multivariable regression analysis was performed to calculate the TTKG means accounting for possible confounding factors, using TTKG as dependent variable and selected variables as covariates. All statistical analyses were performed using the Statistical Package for Social Sciences (SPSS‐PC version 11; SPSS Inc., Chicago, IL).

## Results

As shown in Table 1, the main characteristics of the 25 carriers and 100 noncarriers of the G variant are very similar. There was a 20% lower serum insulin concentration in the carriers than in the noncarriers but this difference did not reach statistical significance. There was no difference in type and amount of antihypertensive treatment between groups (Table 2).

**Table 1:** Characteristics of the subjects according to the glucagon receptor gene polymorphism

	Noncarriers (*N* = 100)	Carriers (*N* = 25)	*P*
Age (y)	57 (56–59)	57 (54–61)	NS
BMI (kg/m^2^)	28 (27–28)	28 (27–29)	NS
Waist circumf. (cm)	98 (97–100)	100 (96–103)	NS
SBP (mm Hg)	136 (133–139)	138 (134–143)	NS
DBP (mm Hg)	89 (87–91)	90 (87–92)	NS
Heart rate (bpm)	67 (66–69)	68 (64–72)	NS
Creat.Clear (ml/min × 1.73 m^2^)	86 (82–90)	88 (81–95)	NS
Serum insulin (mU/L)	9.6 (8.5–10.7)	7.5 (5.9–9.2)	NS (*P* = 0.09) *
Serum glucose (mmol/L)	5.47 (5.24–5.69)	5.48 (4.78–6.17)	NS *

Means (95% CI). *P* from ANOVA for normally distributed variables, and from a Mann–Whitney *U* test for nonnormally distributed variables (*).

**Table 2:** Antihypertensive drug consumption among participants according to the glucagon receptor gene polymorphism (in % of all subjects in each group)

	Noncarriers (*N* = 100)	Carriers (*N* = 25)	*P*
Number of participants treated with antihypertensive drugs	36 (36%)	9 (36%)	NS
Mean number of antihypertensive drugs per subject	0.6	0.6	NS
ACE inhibitors (% of subjects)	18.8	16.7	NS
Angiotensin Receptor Blockers (% of subjects)	6.2	11.1	NS
Calcium channel blockers (% of subjects)	14.1	22.2	NS
Beta‐blockers (% of subjects)	9.4	0	NS
Alpha‐receptor blockers (% of subjects)	0	11.1	NS
Diuretics (% of subjects)	1.6	0	NS

Table 3 displays the main results concerning renal potassium handling according to the glucagon receptor gene polymorphism. There was no difference in serum potassium concentration between the two groups. The mean 24 h potassium excretion rate was also similar in the two groups. However, the urinary concentration of potassium, the urine/serum potassium concentration ratio, and the TTKG were significantly lower by about 20% in the carriers compared to noncarriers. By contrast, there was no difference in urinary sodium concentration. The average urine volume tended to be slightly higher and urine osmolarity slightly lower in the carriers than in the noncarriers, which may suggest a modest urine concentrating defect in the carriers of the mutation.

**Table 3:** Main serum and urine variables according to the glucagon receptor gene polymorphism

** **	Noncarriers (CC)	Carriers (GC)	GC/CC	*P*
Serum K conc. (mmol/L)	4.5 (4.4–4.6)	4.5 (4.3–4.6)	1.00	NS
Urine K conc. (mmol/L)	47 (43–51)	38 (34–43)	0.81	*P* = 0.030
K excretion rate (mmol/d)	68 (64–73)	66 (57–74)	0.97	NS
Urine K / Serum K ratio	10.6 (9.7–11.5)	8.6 (7.6–9.6)	0.81	*P* = 0.034
FE K (%)	11.7 (10.9–12.4)	10.8 (9.5–12.0)	0.92	NS
TTKG	5.0 (4.7–5.2)	4.2 (3.9–4.6)	0.84	*P* = 0.015
Serum urea conc. (mmol/L)	6.2 (5.9–6.5)	6.4 (5.8–7.0)	1.03	NS
Urine urea conc. (mmol/d)	394 (370–419)	440 (387–493)	1.11	NS
Serum Na conc (mmol/L)	140 (139–140)	139 (137–140)	0.99	NS
Urine Na conc. (mmol/L)	137 (128–146)	136 (115–156)	0.99	NS
Na excretion rate mmol/d)	202 (189–214)	228 (197–259)	1.13	NS
Serum osmolarity (mosm/L)	299 (298–301)	298 (296–300)	0.99	NS
Urine volume (L/d)	1.56 (1.46–1.66)	1.72 (1.57–1.87)	1.10	NS
Urine osmolarity (mosm/L)	641 (601–681)	608 (544–673)	0.95	NS

Means (95% CI). *P* from ANOVA (all variables normally distributed)

**Figure 1:**
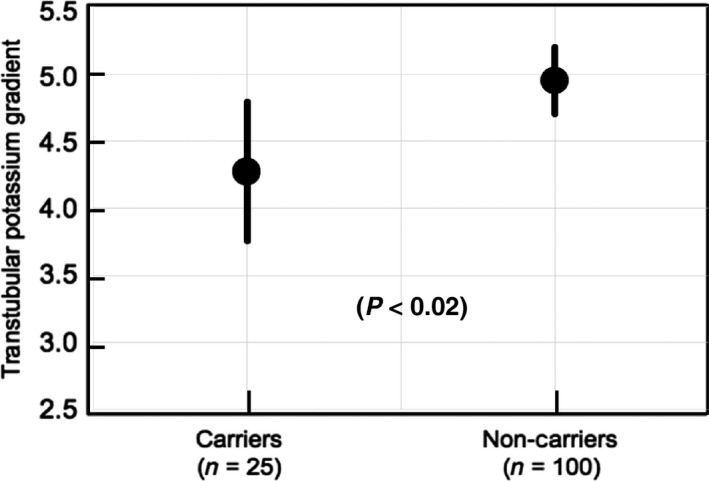
Difference in transtubular potassium gradient between carriers and noncarriers of the GCGR gene polymorphism after multivariate regression analysis accounting for serum insulin and 24‐h sodium and urea excretion rates.

Multivariate regression analysis was used to assess the impact of relevant confounders on the TTKG between‐group difference. After adjustment for serum insulin (that was about 20% lower in the carriers, even if this difference was not significant, see Table 1) and for 24‐h urinary urea and sodium excretion rates (because these other solutes could influence potassium handling), TTKG remained significantly lower (by ≈15 %) in the GC than in the CC participants (Fig. 1): GC = 4.27 (95% CI = 3.76–4.79) versus CC = 4.95 (4.70–5.20) (*P* < 0.02). This model explains about 7% of the overall TTKG variance.

## Discussion

In this study, to determine if glucagon influences the renal handling of potassium, we took advantage of a variant of the glucagon receptor gene that results in a partial loss of function of the glucagon receptor. We compared 25 heterozygous carriers of the mutation (GC) with 100 control subjects without the mutation (CC). The results did not reveal any between‐group difference in serum potassium concentration in a fasting morning blood sample but the urinary potassium concentration was lower by 20% in the carriers of the mutation. The TTKG, which reflects the intensity of potassium secretion in the cortical collecting duct, was also significantly lower in the carriers of the mutation, even after adjustment for possible confounders. The differences observed cannot be accounted for by differences in potassium intake because the 24 h potassium excretion rate, a reliable marker of potassium intake, was similar in both groups.

Epidemiological associations do not allow establishing a causality link between two factors. However, when a phenotypic variable is associated with a genetic alteration leading to a significant functional change (here, the partial loss of function of the glucagon receptor), it represents a Mendelian randomization that allows to draw a causality link between the genotype and the phenotype (Smith and Ebrahim 2003; Didelez and Sheehan 2007; Verduijn et al. 2010). Alleles are randomly allocated during gamete formation and thus the risk for confounding of the genetic variant to the outcome association and for reverse causality is minimized. Thus, the present results do support a causal role for the altered glucagon receptor in the abnormal renal potassium handling.

We had previously observed in rats that the infusion of glucagon increases the excretion of potassium and the TTKG (Ahloulay et al. 1995). The present results in humans are in good agreement with the conclusions of the rat study. Altogether these rat and human results strongly suggest that glucagon plays a role in potassium excretion by stimulating potassium secretion in the distal nephron. The infusion of potassium in conscious dogs was shown to stimulate simultaneously both insulin and glucagon secretion (Santeusanio et al. 1973). It is logical to assume that both hormones may contribute to the regulation of P_K_ in opposite ways, like they do for plasma glucose. An independent regulation of glycemia and P_K_ can be achieved in a combinatory mode as explained previously (Bankir et al. 2016). In the basal state (as seen at wake‐up in early morning) both insulin and glucagon secretion are low as well as most metabolic and excretory functions. An acute carbohydrate intake will selectively increase insulin secretion while a prolonged fast will selectively stimulate glucagon secretion. A meat meal, bringing significant amounts of potassium along with proteins, will increase the secretion of both hormones. These combinatory changes can account for an independent regulation of both plasma glucose and plasma potassium concentrations (Bankir et al. 2016).

This study revealed no between‐group difference in serum potassium concentration. But it should be stressed that the blood sample was taken in the morning after a usual nighttime fast. Plasma glucagon concentration (P_glucagon_) is low in the early morning and rises after each meal (as shown in Fig. 1 of (Velho et al. 1996)). Thus, the early timing of blood collection in this study was not well suited for revealing a possible defect due to the mutated receptor because glucagon's action on its receptors was probably at its lowest level. Apart from the unfavorable timing of blood collection, this study has several other limitations, including the small number of subjects, the fact that only men were studied, that only one TTKG value was available per subject, and that serum (or plasma) glucagon concentration was not measured. It is conceivable that glucagon secretion is reset to a higher level in the carriers of the mutation, thus, possibly attenuating the consequences of the reduced responsiveness of the receptor. To our knowledge, plasma glucagon concentration has not been reported in any of the papers that have studied subjects with this mutation. Aldosterone was unfortunately not available in this study. But even if there was a significant difference between the two groups, it could be interpreted as an adaptative response to the receptor mutation and its functional consequences, because of the Mendelian randomization characteristics of this study (see above).

TTKG has been used in a number of studies in human subjects to reflect the intensity of potassium secretion in the cortical collecting duct. It can be used only if urine Na concentration exceeds 25 mEq/L and urine osmolality exceed plasma osmolality (Choi and Ziyadeh 2008), thus, when vasopressin exerts its antidiuretic action. These conditions are obviously met in this study. The usefullness of TTKG has been documented in several studies in human subjects (West et al. 1986b; Ethier et al. 1990; Rodriguez‐Soriano et al. 1990; Musso et al. 2006).

It is possible to assume that, after each meal, P_K_ should remain higher for a longer time in GC than in CC subjects. In this case, we could also expect a larger difference in the fractional excretion of potassium and in TTKG between the two groups because P_K_ is one of the elements of the formulas used to calculate these variables.

Ideally, the comparison of the two groups should be evaluated by collecting blood and urine after the intake of a standardized protein meal or an infusion of amino acids that are known to significantly increase glucagon secretion (see review in (Bankir et al. 2015)). In order to specifically investigate the consequences of the glucagon receptor mutation on potassium handling, it would be appropriate to compare plasma potassium concentration and TTKG after a standardized oral potassium load in a “potassium tolerance test” similar to the glucose tolerance test. Alternatively, or in addition, it would be interesting to evaluate if subjects treated with selective nonpeptide glucagon receptor antagonists (Shen et al. 2011; Filipski 2015; Kelly et al. 2015) exhibit a higher and more durable rise in plasma potassium after each meal. But in most, if not all studies, blood is taken in the early morning, not after the meals.

In conclusion, this study, although based on a nonoptimal timing of blood sampling, revealed that a partial loss‐of‐function mutation of the glucagon receptor leads to a reduced efficiency of urinary potassium excretion in humans. The fact that glucagon influences renal potassium handling suggests that glucagon might play a role in potassium homeostasis, along with insulin, and in addition to aldosterone. This hypothesis should be further evaluated by clinical investigations involving a standardized protein meal (or an amino acid infusion), and/or a potassium load, comparing plasma potassium and TTKG in subjects with the mutation and control subjects.

## Conflict of Interest

None declared.

## References

[phy213661-bib-0001] Ahloulay, M. , M. Dechaux , K. Laborde , and L. Bankir . 1995 Influence of glucagon on GFR and on urea and electrolyte excretion: direct and indirect effects. Am. J. Physiol. 269:F225–F235. 7653596 10.1152/ajprenal.1995.269.2.F225

[phy213661-bib-0002] Bankir, L. , R. Roussel , and N. Bouby . 2015 Protein‐ and diabetes‐induced glomerular hyperfiltration: role of glucagon, vasopressin, and urea. Am. J. Physiol. Renal Physiol. 309:F2–F23. 25925260 10.1152/ajprenal.00614.2014

[phy213661-bib-0003] Bankir, L. , N. Bouby , B. Blondeau , and G. Crambert . 2016 Glucagon actions on the kidney revisited: possible role in potassium homeostasis. Am. J. Physiol. Renal Physiol. 311:F469–F486. 27194722 10.1152/ajprenal.00560.2015

[phy213661-bib-0004] Barbato, A. , P. Russo , A. Venezia , V. Strazzullo , A. Siani , and F. P. Cappuccio . 2003 Analysis of Gly40Ser polymorphism of the glucagon receptor (GCGR) gene in different ethnic groups. J. Hum. Hypertens. 17:577–579. 12874616 10.1038/sj.jhh.1001591

[phy213661-bib-0005] Cappuccio, F. P. , P. Strazzullo , E. Farinaro , and M. Trevisan . 1993 Uric acid metabolism and tubular sodium handling, Results from a population‐based study.. JAMA 270:354–359. 8315780

[phy213661-bib-0006] Choi, M. J. , and F. N. Ziyadeh . 2008 The utility of the transtubular potassium gradient in the evaluation of hyperkalemia. J. Am. Soc. Nephrol. 19:424–426. 18216310 10.1681/ASN.2007091017

[phy213661-bib-0007] Didelez, V. , and N. Sheehan . 2007 Mendelian randomization as an instrumental variable approach to causal inference. Stat. Methods Med. Res. 16:309–330. 17715159 10.1177/0962280206077743

[phy213661-bib-0008] Ethier, J. H. , K. S. Kamel , P. O. Magner , J. Jr Lemann , and M. L. Halperin . 1990 The transtubular potassium concentration in patients with hypokalemia and hyperkalemia. Am. J. Kidney Dis. 15:309–315. 2321642 10.1016/s0272-6386(12)80076-x

[phy213661-bib-0009] Filipski, K. J. 2015 Small molecule glucagon receptor antagonists: a patent review (2011–2014). Expert Opin. Ther. Pat. 25:819–830. 25828189 10.1517/13543776.2015.1032250

[phy213661-bib-0010] Galletti, F. , A. Barbato , M. Versiero , R. Iacone , O. Russo , G. Barba , et al. 2007 Circulating leptin levels predict the development of metabolic syndrome in middle‐aged men: an 8‐year follow‐up study. J. Hypertens. 25:1671–1677. 17620965 10.1097/HJH.0b013e3281afa09e

[phy213661-bib-0011] Gumz, M. L. , L. Rabinowitz , and C. S. Wingo . 2015 An integrated view of potassium homeostasis. N. Engl. J. Med. 373:60–72. 26132942 10.1056/NEJMra1313341 PMC5675534

[phy213661-bib-0012] Hager, J. , L. Hansen , C. Vaisse , N. Vionnet , A. Philippi , W. Poller , et al. 1995 A missense mutation in the glucagon receptor gene is associated with non‐insulin‐dependent diabetes mellitus. Nat. Genet. 9:299–304. 7773293 10.1038/ng0395-299

[phy213661-bib-0013] Hansen, L. H. , N. Abrahamsen , J. Hager , L. Jelinek , W. Kindsvogel , P. Froguel , et al. 1996 The Gly40Ser mutation in the human glucagon receptor gene associated with NIDDM results in a receptor with reduced sensitivity to glucagon. Diabetes 45:725–730. 8635644 10.2337/diab.45.6.725

[phy213661-bib-0014] Kelly, R. P. , P. Garhyan , E. Raddad , H. Fu , C. N. Lim , M. J. Prince , et al. 2015 Short‐term administration of the glucagon receptor antagonist LY2409021 lowers blood glucose in healthy people and in those with type 2 diabetes. Diabetes Obes. Metab. 17:414–422. 25656305 10.1111/dom.12446

[phy213661-bib-0015] Musso, C. , V. Liakopoulos , R. De Miguel , N. Imperiali , and L. Algranati . 2006 Transtubular potassium concentration gradient: comparison between healthy old people and chronic renal failure patients. Int. Urol. Nephrol. 38:387–390. 16868716 10.1007/s11255-006-0059-5

[phy213661-bib-0016] Rabinowitz, L. 1991 Do splanchnic potassium receptors initiate a kaliuretic reflex? News Physiol. Sci. 6:166–169.

[phy213661-bib-0017] Rodriguez‐Soriano, J. , M. Ubetagoyena , and A. Vallo . 1990 Transtubular potassium concentration gradient: a useful test to estimate renal aldosterone bio‐activity in infants and children. Pediatr. Nephrol. 4:105–110. 2397174 10.1007/BF00858819

[phy213661-bib-0018] Santeusanio, F. , G. R. Faloona , J. P. Knochel , and R. H. Unger . 1973 Evidence for a role of endogenous insulin and glucagon in the regulation of potassium homeostasis. J. Lab. Clin. Med. 81:809–817. 4710366

[phy213661-bib-0019] Shen, D. M. , S. Lin , and E. R. Parmee . 2011 A survey of small molecule glucagon receptor antagonists from recent patents (2006–2010). Expert Opin. Ther. Pat. 21:1211–1240. 21635155 10.1517/13543776.2011.587001

[phy213661-bib-0020] Shiota, D. , T. Kasamatsu , S. A. Dib , A. R. Chacra , and R. S. Moises . 2002 Role of the Gly40Ser mutation in the glucagon receptor gene in Brazilian patients with type 2 diabetes mellitus. Pancreas 24:386–390. 11961492 10.1097/00006676-200205000-00010

[phy213661-bib-0021] Siani, A. , R. Iacone , O. Russo , G. Barba , P. Russo , F. P. Cappuccio , et al. 2001 Gly40Ser polymorphism of the glucagon receptor gene is associated with central adiposity in men. Obes. Res. 9:722–726. 11707539 10.1038/oby.2001.97

[phy213661-bib-0022] Smith, G. D. , and S. Ebrahim . 2003 ‘Mendelian randomization’: can genetic epidemiology contribute to understanding environmental determinants of disease? Int. J. Epidemiol. 32:1–22. 12689998 10.1093/ije/dyg070

[phy213661-bib-0023] Strazzullo, P. , R. Iacone , A. Siani , G. Barba , O. Russo , P. Russo , et al. 2001 Altered renal sodium handling and hypertension in men carrying the glucagon receptor gene (Gly40Ser) variant. J. Mol. Med. (Berl) 79:574–580. 11692154 10.1007/s001090100257

[phy213661-bib-0024] Tonolo, G. , M. G. Melis , M. Ciccarese , G. Secchi , M. M. Atzeni , M. Maioli , et al. 1997 Physiological and genetic characterization of the Gly40Ser mutation in the glucagon receptor gene in the Sardinian population. The Sardinian Diabetes Genetic Study Group. Diabetologia 40:89–94. 9028723 10.1007/s001250050647

[phy213661-bib-0025] Velho, G. , K. F. Petersen , G. Perseghin , J. H. Hwang , D. L. Rothman , M. E. Pueyo , et al. 1996 Impaired hepatic glycogen synthesis in glucokinase‐deficient (MODY‐2) subjects. J. Clin. Invest. 98:1755–1761. 8878425 10.1172/JCI118974 PMC507613

[phy213661-bib-0026] Verduijn, M. , B. Siegerink , K. J. Jager , C. Zoccali , and F. W. Dekker . 2010 Mendelian randomization: use of genetics to enable causal inference in observational studies. Nephrol. Dial. Transplant. 25:1394–1398. 20190244 10.1093/ndt/gfq098

[phy213661-bib-0027] West, M. L. , O. Bendz , C. B. Chen , G. G. Singer , R. M. Richardson , H. Sonnenberg , et al. 1986a Development of a test to evaluate the transtubular potassium concentration gradient in the cortical collecting duct in vivo. Miner. Electrolyte Metab. 12:226–233. 3762509

[phy213661-bib-0028] West, M. L. , P. A. Marsden , R. M. Richardson , R. M. Zettle , and M. L. Halperin . 1986b New clinical approach to evaluate disorders of potassium excretion. Miner. Electrolyte Metab. 12:234–238. 3762510

[phy213661-bib-0029] Youhanna, S. , L. Bankir , P. Jungers , D. Porteous , O. Polasek , M. Bochud , et al. 2017 Validation of surrogates of urine osmolality in population studies. Am. J. Nephrol. 46:26–36. 28586769 10.1159/000475769 PMC6080694

